# Lunasin Attenuates Obesity-Associated Metastasis of 4T1 Breast Cancer Cell through Anti-Inflammatory Property

**DOI:** 10.3390/ijms17122109

**Published:** 2016-12-15

**Authors:** Chia-Chien Hsieh, Chih-Hsuan Wang, Yu-Shan Huang

**Affiliations:** Department of Human Development and Family Studies, National Taiwan Normal University, Taipei 10610, Taiwan; joyce.walawala@gmail.com (C.-H.W.); z83526@hotmail.com (Y.-S.H.)

**Keywords:** adipocyte, breast cancer, inflammation, lunasin, metastasis

## Abstract

Obesity prevalence is increasing worldwide and is accompanied by low-grade inflammation with macrophage infiltration, which is linked with a poorer breast cancer prognosis. Lunasin is a natural seed peptide with chemopreventive properties and multiple bioactivities. This is the first study to explore the chemopreventive effects of lunasin in the obesity-related breast cancer condition using 4T1 breast cancer cells, 3T3-L1 adipocytes, and conditioned media. An obesity-related environment, such as leptin-treatment or adipocyte-conditioned medium (Ad-CM), promoted 4T1 cell proliferation and metastasis. Lunasin treatment inhibited metastasis of breast cancer cells, partially through modestly inhibiting production of the angiogenesis-mediator vascular endothelial growth factor (VEGF) and significantly by inhibiting secretion in the Ad-CM condition. Subsequently, two adipocytes inflammation models, 3T3-L1 adipocytes were stimulated by tumor necrosis factor (TNF)-α, and RAW 264.7 cell-conditioned medium (RAW-CM) was used to mimic the obese microenvironment. Lunasin significantly inhibited interleukin (IL)-6 and macrophage chemoattractant protein (MCP)-1 secretion by TNF-α stimulation, and MCP-1 secretion in the RAW-CM model. This study highlights that lunasin suppressed 3T3-L1 adipocyte inflammation and inhibited 4T1 breast cancer cell migration. Interestingly, lunasin exerted more effective anti-metastasis activity in the obesity-related condition models, indicating that it possesses anti-inflammatory properties and blocks adipocyte-cancer cell cross-talk.

## 1. Introduction

Obesity is currently one of the major public health problems, and its presence is associated with many chronic diseases, including diabetes, atherosclerosis, and cancer. The prevalence of overweight and obese individuals is continually increasing worldwide [1]. In the obese microenvironment, adipose tissue is characterized by hyper-accumulation of lipids accompanied by low-grade inflammation. Various types of immune cells infiltrate the adipose tissue, and are associated with increased secretion of pro-inflammatory adipokines and cytokines such as leptin, interleukin (IL)-6, macrophage chemoattractant protein (MCP)-1 and tumor necrosis factor (TNF)-α [2,3,4]. Obesity increases the risk and progression for many cancers, especially breast cancer, which is the most common cancer in women today. The development and prognosis of breast cancer have been consistently shown to be negatively correlated to obese status [5,6].

Cancer develops when neoplastic transformed cells escape from immune responses, leading to transformation and alteration of tissue structures [7]. A growing body of evidence supports this process, which is known as cancer immunoediting, suggesting an intact immune system prevents/controls neoplasia [8]. Inflammation is a complex process caused by pathogens or diseases. Interestingly, chronic inflammation has been demonstrated to be present in about 15%–20% of developed malignancies [9], and is accompanied by oxidative stress [10]. In the microenvironment, recruitment of immune cells such as macrophages that generate a series of pro-inflammatory mediators bridged innate immune response with adaptive immunity, which contributes to neoplastic transformation, thereby increasing tumor invasion and metastasis [11]. This tumor microenvironment has been revealed to have a varied cellular composition, including tumor cells, stromal fibroblasts, endothelial cells, and infiltrating leukocytes and macrophages [7]. In this environment, several biological mediators are secreted to support a microenvironment favorable for neoplasia, and to participate in angiogenesis involved in cell proliferation, migration, and remodeling of endothelial cells [12].

Lunasin is a natural peptide with 43 amino acids; it was first identified in the soybean [13] and other grains and herbal plants [14]. The effect of lunasin on chronic diseases, including cancer, cardiovascular disease, and immunological disorders, has been extensively studied [14]. Lunasin exerts antioxidant and anti-inflammatory properties [15] which may contribute to its chemopreventive response. The anti-inflammatory properties of lunasin were revealed by its ability to inhibit pro-inflammatory mediator secretion by lipopolysaccharide (LPS)-stimulated RAW 264.7 cells [15]. Subsequently, the ability of lunasin to block nuclear factor (NF)-κB signaling pathways has also been demonstrated [16,17], which was found to be via downregulation of Akt phosphorylation and p65 protein expression in activated human THP-1 macrophages [18]. These findings indicate that the anti-inflammatory properties of lunasin could contribute to its protective effects against several related disorders. Investigation into phytochemical chemoprevention capabilities is encouraged for the development of promising natural agents to prevent and/or cure cancer progression without additional side effects.

The pathological and physiological mechanisms linking obesity and breast cancer are currently attracting the attention of the scientific community. There is still limited evidence in this field, and there are no data showing the effects of lunasin in a model of obesity. In the present study, we investigated the potentially chemopreventive and anti-inflammatory bioactivities of lunasin using 4T1 murine breast cancer cells and 3T3-L1 adipocytes. A comprehensive understanding of the mechanism of action is required in order to be able to propose a lunasin-rich diet as an auxiliary therapy in the treatment of obesity-related breast cancer.

## 2. Results

### 2.1. Leptin and Adipocyte-Conditioned Medium (Ad-CM) Stimulated 4T1 Breast Cancer Cell Proliferation

**Figure 1:**
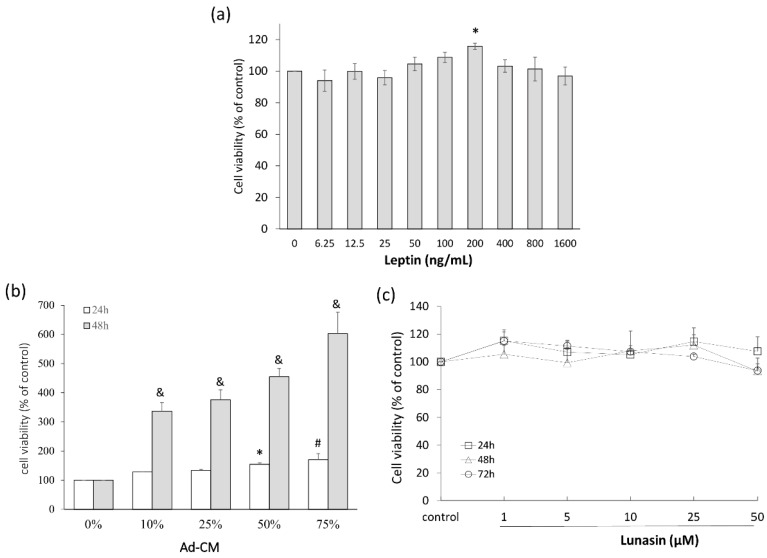
Cell growth of breast cancer 4T1 cells treated with leptin and adipocyte-conditioned medium (Ad-CM). (**a**) Cells were treated with different concentrations of recombinant mouse leptin for 24 h, and the viability of cells was analyzed; (**b**) Cells were treated with different percentages of Ad-CM for 24 and 48 h, and the viability of cells was analyzed; (**c**) Cells were treated with different dosages of lunasin for 24, 48, and 72 h, and the viability of cells was analyzed by methylthiazole tetrazolium (MTT) assay. Data are shown as mean ± standard error of the mean (SEM). Statistical analysis was tested by one-way ANOVA and then Fisher’s least significant difference (LSD) test; significant differences are presented as * *p* < 0.05, # *p* < 0.01, or & *p* < 0.005 versus control group.

To investigate the effect of obesity-related mediators on 4T1 breast cancer cell growth, recombinant leptin and Ad-CM were used to mimic the physiological conditions of obesity, and cell viability was determined by methylthiazole tetrazolium (MTT) assay. 4T1 cells were treated with different doses of leptin, and the 200 ng/mL dose significantly increased cell proliferation (Figure 1a). In the other model, 4T1 cells were cultured in 0%, 10%, 25%, 50%, or 75% Ad-CM for 24 or 48 h; the control cell proliferation, means 0% Ad-CM, was set as the baseline. A dose-dependent increase was observed in Ad-CM-treated 4T1 cells relative to that of the control group. Cell number increased by 337% (*p* = 0.002), 375% (*p* = 0.001), 445% (*p* < 0.001), and 602% (*p* < 0.001) relative to that of control cells when treated with 10%, 25%, 50%, and 75% Ad-CM, respectively (Figure 1b). We then investigated whether lunasin treatments affected cell proliferation using an MTT assay to determine cell viability. Lunasin treatment for 24, 48, or 72 h did not affect the cell viability (Figure 1c).

### 2.2. Lunasin Inhibited 4T1 Cell Metastasis and Vascular Endothelial Growth Factor (VEGF) Production

**Figure 2:**
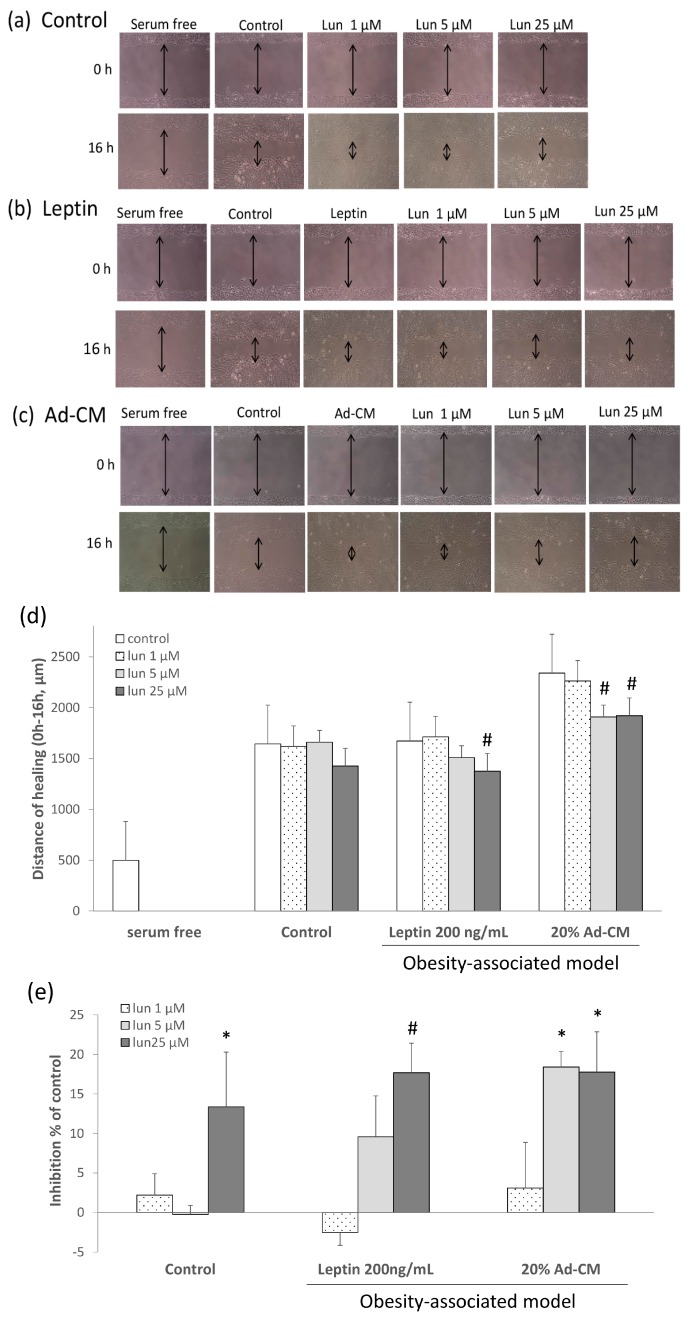
Effect of lunasin on breast cancer 4T1 cell metastasis. Migration patterns were observed in the scraped area of 4T1 cells treated with or without lunasin for 16 h and incubated under three conditions: (**a**) Fresh medium-5% fetal bovine serum/Dulbecco’s modified Eagle’s medium (FBS/DMEM), the mark of double arrows was present the distance of healing in the pictures; (**b**) Leptin at 200 ng/mL in 5% FBS/DMEM; (**c**) 20% Ad-CM in 5% FBS/DMEM; (**d**) The distance of migrated cells was quantified by manual counting under the microscope scale; (**e**) Migrated cells were quantified by manual counting under the microscope and presented as a percentage of inhibition relative to that of control (control as 0%). Data are shown as mean ± SEM. Statistical analysis was tested by one-way ANOVA and then Fisher’s LSD test; significant differences are presented as * *p* < 0.05, or # *p* < 0.01 versus control group.

To explore the effects of lunasin on 4T1 cell metastasis, cell behavior was analyzed using wound healing, as shown in Figure 2. Cells cultured in serum free media without cell proliferation after 16 h were used as the negative control. In the 5% fetal bovine serum/Dulbecco’s modified Eagle’s medium (FBS/DMEM) complete medium, as positive control, lunasin didn not affect the wound healing (Figure 2a). The complete medium was replaced by 200 ng/mL leptin (Figure 2b) or 20% Ad-CM (Figure 2c) to mimic the breast cancer cell-adipocyte microenvironment. Lunasin delayed post-scratch healing relative to the positive control in a concentration-dependent manner after a 16 h treatment under adipocyte-associated models.

Migration distance into the scratch wound was quantified by manual counting of the microscope scale after 16 h of treatment (Figure 2d). In the positive control, the scratch was almost fully healed by 24 h post-injury (data not shown). In the leptin model, treatment with 25 µM lunasin significantly delayed wound healing (*p* = 0.0051). In the Ad-CM model, which more closely mimics physiological conditions, lunasin treatment at 5 and 25 µM doses significantly decreased 4T1 cell migration after scratch (*p* = 0.0043, *p* = 0.0051, respectively) (Figure 2d). Results are shown as a percentage relative to the control group, and indicate that 25 µM lunasin inhibited cell migration in complete medium and leptin models by 13.3% and 17.7%, respectively (*p* = 0.042, *p* = 0.005). Likewise, 5 and 25 µM lunasin doses inhibited migration in the adipocyte-related environment (Ad-CM) by 18.4% and 17.7%, respectively, relative to that of the control (*p* = 0.011, *p* = 0.012; Figure 2e). These data suggest that lunasin treatments induced significant inhibition of cell migration in the obesity-related models.

**Figure 3:**
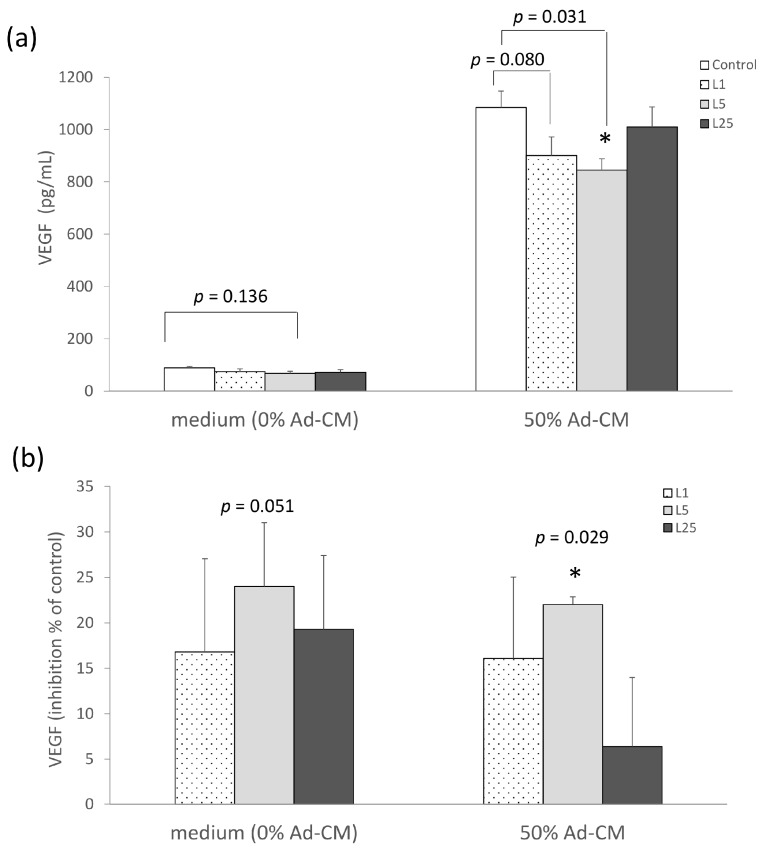
Effect of lunasin treatment on cytokine vascular endothelial growth factor (VEGF) production in 4T1 breast cancer cells. (**a**) Effect of lunasin on the amount of VEGF in 4T1 cells in complete medium or 50% Ad-CM; (**b**) Effect of lunasin on the amount of VEGF in 4T1 cells cultured in complete medium or 50% Ad-CM is presented as a percentage of inhibition relative to control (control as 0%). The VEGF production in supernatant obtained from 48 h culture medium was measured by enzyme-linked immunosorbent assay (ELISA). Data are shown as mean ± SEM. Statistical analysis was tested by one-way ANOVA and then Fisher’s LSD test; significant differences are presented as * *p* < 0.05 versus control group.

We then continued by investigating whether lunasin treatment affected the production of angiogenesis-related cytokine. Cultured supernatants were collected for VEGF assay. The concentration of VEGF in the 5 µM lunasin treated group trended towards a decrease compared to the control group (*p* = 0.136) in complete medium (0% Ad-CM). This level was significantly decreased in the 5 µM lunasin treatment compared to that of the control group in 50% Ad-CM (*p* = 0.031) (Figure 3a). In addition, the inhibited percentage of VEGF in the 1, 5, and 25 µM lunasin-treated groups was 17%, 24%, and 19% lower, respectively, than in the control group (*p* = 0.147, *p* = 0.051, *p* = 0.103, respectively; Figure 3b). In the 50% Ad-CM model, the inhibited percentage in the 1, and 5 µM lunasin treatment was 16%, and 22% lower, respectively, compared to that of the control group (*p* = 0.088, *p* = 0.029, respectively; Figure 3b). These data suggest that lunasin tends to inhibit production of the pro-angiogenesis cytokine VEGF, and this suppressive property was significantly effective in the Ad-CM model, resulting in the retardation of the migration of 4T1 breast cancer cells.

### 2.3. Lunasin Did Not Effect 3T3-L1 Adipocytes Differentiation and Lipid Accumulation

To investigate whether lunasin affects cell viability involved lipid accumulation during the adipocyte differentiation process, we examined lipid accumulation in lunasin-treated 3T3-L1 cells. Treatment with multiple doses of lunasin in 3T3-L1 cells had no effect on cell viability (Figure S1a). No differences in adipocyte morphology were observed at the end of cell differentiation (data not shown). Adipocyte lipid droplets were stained with Oil Red O to confirm fat accumulation, which was unaffected by lunasin treatment (Figure S1b).

### 2.4. Lunasin Inhibited Pro-Inflammatory Adipokine Secretion in 3T3-L1 Adipocytes

**Figure 4:**
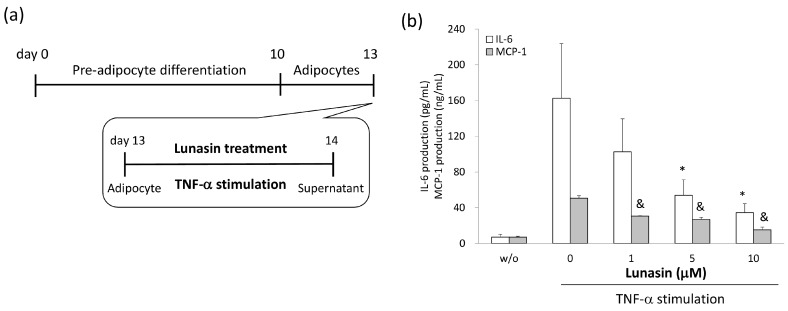
Effect of lunasin on interleukin (IL)-6 and macrophage chemoattractant protein (MCP)-1 secretion in 3T3-L1 adipocytes stimulated by tumor necrosis factor (TNF)-α. (**a**) Inflammatory activation of 3T3-L1 adipocytes was achieved by stimulation with TNF-α, and cells were treated with lunasin at the same time for 24 h; (**b**) IL-6 and MCP-1 production in supernatant was measured by ELISA; Correlation of lunasin dosage and cytokine quantity is presented in the Table 1. Data are shown as mean ± SEM. Statistical analysis was tested by one-way ANOVA and then Fisher’s LSD test; significant differences are presented as * *p* < 0.05 or & *p* < 0.005 versus control group.

**Figure 5:**
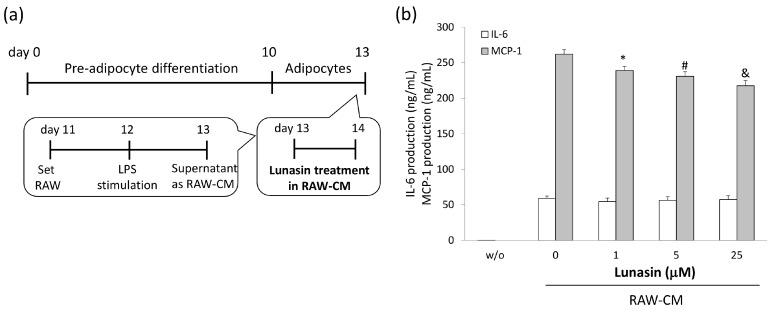
Effect of lunasin on MCP-1 and IL-6 secretion in 3T3-L1 adipocytes stimulated by RAW 264.7 conditioned medium (RAW-CM). (**a**) Inflammatory activation of 3T3-L1 adipocytes was achieved by stimulation with RAW-CM, cells were treated with lunasin for 24 h; (**b**) IL-6 and MCP-1 production in supernatant was measured by ELISA; Correlation of lunasin dosage and cytokine quantity is presented in the Table 2. Data are shown as mean ± SEM. Statistical analysis was tested by one-way ANOVA and then Fisher’s LSD test; significant differences are presented as * *p* < 0.05, # *p* < 0.01, or & *p* < 0.005 versus control group.

To determine the anti-inflammatory properties of lunasin in 3T3-L1 adipocytes, levels of relevant mediators in the cultured supernatants were analyzed. Inflammatory conditions were induced in adipocytes using stimulation with TNF-α or RAW 264.7 cell-conditioned medium (RAW-CM), and adipocytes were simultaneously treated with various concentrations of lunasin (Figure 4 and Figure 5). MCP-1 and IL-6 production were increased in TNF-α and RAW-CM stimulated cells compared to that in unstimulated cells. In the TNF-α stimulated model, lunasin treatments significantly decreased IL-6 and MCP-1 production in mature adipocytes in a dose-dependent manner (*p* < 0.05, Figure 4a,b). Cells treated with 1, 5, and 10 µM lunasin presented significantly decreased IL-6 secretion (34.5%, 63.2% (*p* = 0.036), and 73.8% (*p* = 0.016)) and MCP-1 secretion (39.5% (*p* = 0.001), 47.0% (*p* = 0.001), and 70.3% (*p* < 0.001)) compared to untreated controls, respectively. These data showed a potent negative correlation between lunasin dosage and IL-6 production (*p* = 0.029, *r* = −0.545), and between lunasin dosage and MCP-1 production (*p* = 0.006, *r* = −0.861; Figure 4c).

In the other model, 3T3-L1 adipocytes were cultured with RAW-CM, simulating an obesity-model microenvironment where macrophage infiltration was present (Figure 5a). Cells cultured with 20% RAW-CM and treated with 1, 5, and 10 µμM lunasin showed significantly decreased MCP-1 secretion (90.2% (*p* = 0.032), 89.1% (*p* = 0.006), and 83.4% (*p* < 0.001)) compared to controls, respectively (Figure 5b). However, lunasin treatment did not affect IL-6 secretions in this model. These data were consistent with those obtained using the TNF-α stimulation model and showed a strongly negative correlation between lunasin dosage and MCP-1 production (*p* = 0.006, *r* = −0.651; Figure 5c). These results indicate that lunasin treatment inhibited inflammatory mediator production in the inflammatory adipocyte models, which may contribute significantly to the suppression of obesity-related breast cancer development.

**Figure 6:**
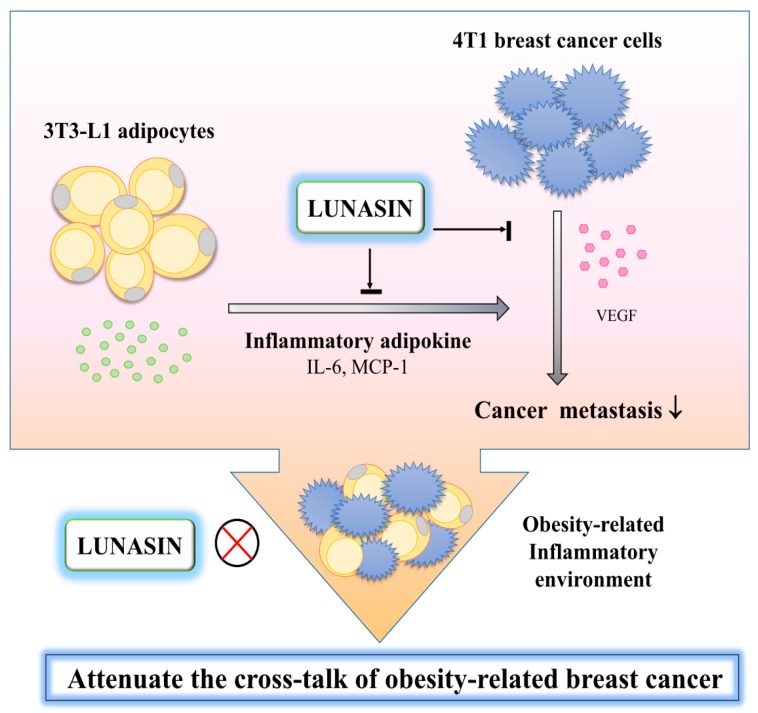
Schematic displaying a possible mechanism by which lunasin attenuates adipocyte inflammation and obesity-related breast cancer cell metastasis. In 3T3-L1 cells, lunasin treatment inhibited the inflammatory adipokine IL-6 and MCP-1 production from adipocytes. In 4T1 cells, lunasin inhibited VEGF production and cell migration. Lunasin significantly inhibited the metastasis of 4T1 cells, especially in the obesity-related microenvironment. These phenomena can be partially attributed to the anti-inflammatory properties of lunasin, as well as its ability to blunt the cross-talk present in this combined system. The arrows denote changes that were observed due to lunasin treatment.

A schematic of the potential mechanisms of action of lunasin, based on the information obtained in this study, is proposed in the Figure 6.

## 3. Discussion

Accumulating evidence has revealed that obesity promotes the incidence and mortality in multiple cancers such as breast cancer [5,6]. The chronic low grade inflammation that accompanies the obese physiological state promotes the infiltration of various immune cells into adipose tissue, cultivating a neoplasia-favorable environment and triggering tumorigenesis [2,3,4]. The chemopreventive properties of lunasin have been demonstrated by both in vitro and in vivo studies [14].

In the present study, the results demonstrated that an obesity-related environment, as simulated by leptin stimulation or adipocyte-conditioned medium incubation, promotes 4T1 breast cancer cell proliferation. However, lunasin treatment inhibited 4T1 cell migration, likely through modestly inhibiting the production of the angiogenesis-mediator VEGF. Subsequently, 3T3-L1 adipocytes were subjected to an inflammatory microenvironment to clarify the mechanisms in obesity-related breast cancer pathogenesis. Cells were stimulated by TNF-α or macrophage-conditioned medium to mimic an obesity-related environment where macrophage infiltration is prevalent. Lunasin treatment significantly inhibited the secretion of the inflammatory adipokines IL-6 and MCP-1 in both inflammation models. This study was the first to highlight that lunasin inhibited the inflammation of 3T3-L1 adipocytes, significantly contributing to blunting the cross-talk present in the obesity-related microenvironment and, thus, suppressing the metastasis of 4T1 cells.

The potential mechanisms linking obesity and breast cancer are of increasing interest to scientists. Growing evidence has shown that obesity promotes mammary tumor progression and uncontrolled growth in obese experimental animal models [6,19], regardless of whether obesity was genetically-induced or diet-induced [20]. In human breast cancer MCF-7 and MDA-MB-231 cells, leptin treatment promoted VEGF secretion and increased cell proliferation and migration compared to controls, whereas treatment with adiponectin exerted the opposite effect [21]. These data indicate that obesity enhances breast tumorigenesis both in vivo and in vitro.

Adequate nutrients and oxygen are essential for tumor growth. Angiogenesis, a rapid and widespread formation of blood vessels, is required for tumor growth and is a hallmark of cancer development. VEGF is one of the lead pro-angiogenic factors in a tumor system, and plays an important role in the tumor microenvironment by supporting tumor cells and surrounding cells [22]. Therefore, anti-angiogenic agents targeting VEGF have been cautiously examined recently in ongoing clinical trials, either combined with other standard protocols or as neo-adjuvant therapy [23]. In this study, lunasin treatment inhibited 4T1 cell metastasis partially owing to the inhibition of VEGF secretion, and this phenomenon was seen particularly in groups treated with leptin or Ad-CM. In our previous studies, lunasin exerted anti-proliferative effects on MDA-MB-231 cells both in vitro and as part of a breast cancer xenograft in vivo model [24,25]. Recently, Jiang et al. have demonstrated that lunasin suppressed the metastasis of MCF-7 and MDA-MB-231 cells via downregulation of matrix metalloproteinase (MMP)-2/-9 expression, which was associated with focal adhesion kinase (FAK)/Akt/extracellular signal-regulated kinases (ERK) and NF-κB signaling [26], suggesting this metastasis inhibition effect of lunasin might be mediated by both integrin and inflammatory pathways.

Multiple immune cells, such as macrophages, infiltrate into the tumor and adipose tissue microenvironments and cultivate a favorable environment for neoplastic cells due to alterations of the local cytokines profile, such as angiogenesis factors (i.e., epidermal growth factor (EGF)) and VEGF and inflammatory mediators (i.e., IL-6, TNF-α, leptin). In obese subjects, increasing levels of circulating VEGF accompanied tumoral expression, which was linked to cancer patients with a poor prognosis [12,27]. This increase of VEGF was also shown in the tumors of obese mice [20]; several inflammatory adipokines, which were increased with obesity, contributed to breast carcinogenesis [21]. Viana [28] have revealed that 4T1 tumor bearing mice showed higher levels of MCP-1, VEGF, and TNF-α in serum compared to controls, suggesting that these mediators might respond to breast tumor growth. Based on this evidence, the 4T1 metastasis inhibition effect of lunasin might be mediated by VEGF regulation and inflammatory pathways.

Physiological inflammatory mediators are potential biomarkers for cancer malignancy. Therefore, developing novel agents against these mediators could prove to be a promising therapeutic route for related diseases [29]. In an in vitro model of obesity, 3T3-L1 adipocytes stimulated by LPS and RAW-CM presented increased IL-6 gene expression, as well as TNF-α and IL-6 cytokine production, but decreased peroxisome proliferator-activated receptor (PPAR)γ gene levels [30]. In this study, lunasin treatments downregulated IL-6 and MCP-1 secretion in 3T3-L1 cells stimulated by TNF-α, suggesting that lunasin exerted anti-inflammatory properties in adipocytes. However, lunasin decreased MCP-1 secretion, but did not affect IL-6 levels in the RAW-CM model. The IL-6 level of the control group was more than 300 times higher in the RAW-CM model compared to that in the TNF-α model. These results indicate that lunasin may not be able to regulate high amounts of IL-6 when increased suddenly in this study. Otherwise, the MCP-1 level was significantly inhibited by lunasin treatment probably due to the MCP-1 level of control group was just five times higher in RAW-CM model, and lunasin still exerted its anti-inflammatory property. MCP-1, also known as CCL2, is a member of the chemokine superfamily and promotes the recruitment and activation of monocytes during acute inflammation and angiogenesis. The levels of MCP-1 and IL-6 in the circulation of obese subjects were often found to be higher than the levels present in healthy animals [2]. Evidence indicated that MCP-1 and IL-6 could assist with macrophage infiltration during inflammation and then promote tumorigenesis, suggesting that lower levels of these adipokines may work against neoplastic disease development [31]. LPS stimulation has been shown to induce promotion of macrophage activation, phagocytosis, and CCL2 secretion by 4T1 cells, indicating an enhanced macrophage-mediated inflammation in breast cancer cells [32].

Lunasin is a natural seed peptide with multiple bioactive properties, such as inflammation reduction, cancer prevention, cholesterol lowering, and immune regulation [14], and has now been sold as a branded ingredient. Lunasin acts against digestive enzyme proteolysis by naturally-occurring protease inhibitors, and resists food processing as it still exerted its bioactivities both in vitro and in vivo [14,24,33,34]. After gastrointestinal digestion, lunasin has been detected in healthy volunteers after receiving 50 g soy protein, and appeared in plasma at concentrations 50 to 111 ng/mL showing an approximately 4.5% absorptive rate [35]. These findings obtained from bioavailability and bioactivity studies support that the intake of lunasin-enriched foods in daily diets is safe and has health benefits. Galvez et al. have demonstrated that lunasin was involved in decreasing the gene expression of hepatic 3-hydroxy-3-methyl-glutaryl (HMG) CoA reductase in HepG2 cells and increasing the expression of the low density lipoprotein (LDL) receptor [36,37]. Moreover, lunasin intervention repressed THP-1 macrophage inflammation by interacting with endocytosis pathways [38]. These studies supported that lunasin may contribute to prevent the cardiovascular disease procession. Furthermore, lunasin has also demonstrated its immunoregulatory activity in other inflammatory diseases; it modulates allergic airway inflammation through increased regulatory T cells in murine models of asthma [39] and reduces immune mediators in cultured synovial fibroblasts from patients [40]. Interestingly, these findings indicated that the anti-inflammatory properties of lunasin could make it a promising agent against several related disorders.

Breast cancer progression and metastasis are tightly linked with cytokine secretion and effects [7,8,9,11]. An exhaustive understanding of the action mechanisms linking obesity, inflammation, and cancer may afford an opportunity to develop accurate strategies to attenuate the negative impacts of obesity. To date, the body of knowledge available is still inadequate and further research is required to demonstrate the efficacy of promising agents in obesity-related breast cancer treatments. In the present study, lunasin, a natural compound, not only weakened the inflammation of 3T3-L1 adipocytes, but also inhibited the migration of 4T1 breast cancer cells. This metastasis suppression was even effective within an obesity-related microenvironment, likely due to the anti-inflammatory properties of lunasin resulting in the blockade of adipocyte-cancer cell cross-talk. Further studies will need to focus on determining more special mediators in conditions of adipocytes, and evaluating the expression and phosphorylation of markers is needed to completely elucidate the mechanism of action. In addition, an animal study should be conducted to confirm the effect and safety of lunasin in vivo.

## 4. Materials and Methods

### 4.1. Cell Culture and Reagents

4T1 murine breast cancer cells were purchased from the American Type Culture Collection (ATCC; Manassas, VA, USA) and RAW 264.7 macrophage cells were kindly provided by Tsai of the National Taiwan Normal University (Taipei, Taiwan). 4T1 is a highly metastatic breast cancer cell line derived spontaneously from a BALB/c mouse, and is a model of a triple-negative breast cell line. This type of breast cancer cell has more challenge during the medical therapy. Mouse 4T1 and RAW 264.7 cells were maintained in Dulbecco’s modified Eagle’s medium (DMEM; Caisson, Smithfield, UT, USA) containing 10% fetal bovine serum (FBS; Genedirex, Las Vegas, NV, USA) with 1% penicillin/streptomycin/amphotericin B (Caisson). Mouse 3T3-L1 fibroblasts were purchased from the Bioresource Collection and Research Center (BCRC; Hsinchu, Taiwan), and were cultured in DMEM containing 10% bovine serum (BS; Gibco, Grand Island, NY, USA) in a 37 °C incubator containing 5% CO_2_ with humidification.

Lunasin was synthesized by Chengdu KaiJie Bio-Pharmaceutical (Chengdu, China) with the sequence: SKWQHQQDSCRKQLQGVNLTPCEKHIMEKIQGRGDDDDDDDDD. The purity of this peptide was greater than 95% and the peptide was stored at −20 °C. The concentrations of lunasin used in this study referred to recently published articles, and this range of doses did not affect the viability and morphology of normal cells [14,15,16,17,18].

### 4.2. 3T3-L1 Fibroblast Differentiation to Adipocytes

3T3-L1 pre-adipocytes were seeded at 3 × 10^4^ cells per well in 24-well plates (Becton Dickinson, Franklin Lakes, NJ, USA) in 10% FBS/DMEM. After 2 days, differentiation was induced in cells via incubation with medium I (25 mM glucose, 0.5 mM 3-isobutyl-1-methylxanthine (Sigma Aldrich, St. Louis, MO, USA), 0.2 µM dexamethasone (Sigma Aldrich), 10 µg/mL insulin (Sigma Aldrich) in 10% FBS/DMEM) for 4 days. Medium I was then replaced by medium II (25 mM glucose, 10 µg/mL insulin, and 10% FBS/DMEM), which was replaced with fresh medium II every 3 days to trigger adipocyte maturation by approximately day 13. These mature adipocytes were then used for further experimentation.

#### Quantification of 3T3-L1 Lipid Accumulation

During the differentiation period, 3T3-L1 cells were treated with different doses of lunasin until the end. Cells were washed using phosphate buffered saline (PBS), fixed with 10% paraformaldehyde at 25 °C for 30 min, and then stained with Oil Red O (Sigma Aldrich) solution for 40 min. Cultured cells containing lipid droplets were visualized using a microscope at 200× magnification (Nikon, Tokyo, Japan), and then Oil Red O dye was solubilized using 2-propanol and quantified via spectrophotometry by using a microplate reader (BioTek, Winooski, VT, USA) to measure absorbance at 500 nm.

### 4.3. Generation of Conditioned Media

The methods of conditioned media generation were adapted from a previous study [41].

Adipocyte-conditioned medium (Ad-CM): After 3T3-L1 adipocytes differentiated for 12 days, the medium was switched to DMEM for 24 h. At the end of this period, the supernatant was collected as adipocyte-conditioned medium (Ad-CM) for subsequent in vitro studies.

RAW 264.7 cell-conditioned medium (RAW-CM): RAW 264.7 cells were seeded in 24-well plates at a density of 5 × 10^5^ per well in 10% FBS/DMEM overnight, and then cells were incubated with 1% FBS/DMEM and activated by LPS (100 ng/mL, Sigma Aldrich) for 24 h. Thereafter, the supernatant was collected as RAW 264.7 cell-conditioned medium (RAW-CM).

### 4.4. Cell Metastasis Assay

A wound healing assay was used to investigate the migration of breast cancer cells. 4T1 cells were seeded at a density of 1 × 10^5^ cells per well in 24-well plates overnight until cells reached 80% confluence in 5% FBS/DMEM. The cell monolayer was scratched carefully using a 20 µL pipette tip, and then the medium was switched to contain either 5% FBS/DMEM complete medium, 200 ng/mL recombinant mouse leptin (R&D, Minneapolis, MN, USA), or 20% Ad-CM. These various media were simultaneously either treated or untreated with various doses of lunasin. Imaging was performed using a microscope at 100× magnification and a WS500 camera (Whited, Taoyuan, Taiwan) at 0, 16, and 24 h post-scratch. Migrated cells were quantified by manual counting of microscope scale. The results were also calculated as a percentage relative to the control group. This assay was replicated at least three times for each experimental group.

### 4.5. Cell Viability Assay

#### 4T1 Cells Cultured in Ad-CM

To test the effect of Ad-CM on 4T1 cell growth, 4T1 cells were cultured for 24 and 48 h in different ratios of Ad-CM conditioned medium to mimic the breast cancer cell-adipocyte microenvironment. Both Ad-CM and the unconditioned media contained 1% FBS/DMEM to provide a base level of nutrition for cellular function.

4T1 and 3T3-L1 cells were seeded at densities of 1 × 10^3^ and 3 × 10^4^ per well, respectively, in 96-well plates (Becton Dickinson) and treated with various concentrations of lunasin for 24, 48, and 72 h. Methylthiazole tetrazolium (0.5 mg/mL, MTT; Sigma Aldrich) solution was then added to the cells for 3 h at 37 °C after the completion of the lunasin treatment period. The supernatant was aspirated and mixed with dimethyl sulfoxide for 20 min to solubilize formazan crystals. Spectrophotometry was performed and the absorbance (A) at 540 nm was measured using a microplate reader (BioTek). Cell viability was expressed using the formula ((Asample − Ablank)/(Acontrol − Ablank)) × 100. The results were calculated as a percentage of the control, which was considered as 100%.

### 4.6. Determination of Inflammatory Cytokine Production by Enzyme Linked Immunosorbent Assay (ELISA)

4T1 and 3T3-L1 cells were plated at 1.5 × 10^5^ cells per well in a 48-well plate and 3 × 10^4^ cells in a 24-well plate, respectively, overnight. Cells were treated with various doses of lunasin for 24 h. The supernatants were collected and cytokine concentrations were analyzed using the enzyme-linked immunosorbent assay (ELISA) method in accordance with the manufacturer’s instructions (R&D). Cytokines examined included vascular endothelial growth factor (VEGF), IL-6, and MCP-1. The cultured supernatants were diluted 1 to 4 times for VEGF, 1 to 400 times for IL-6, and 500 to 8000 times for MCP-1 analyses. In brief, capture antibodies, cultured supernatants, detection antibodies, and streptavidin conjugated horseradish-peroxidase were processed in order on the plate. Finally, tetramethylbenzidine was used as a color subtract. The absorbance was measured using a microplate reader (BioTek), and the concentration was calculated according to the standard.

### 4.7. Adipocyte Inflammatory Models

3T3-L1 cells were seeded at 3 × 10^4^ cells per well in a 24-well plate for induced differentiation into adipocytes. On day 13, 3T3-L1 adipocytes were treated with various doses of lunasin and stimulated with either 2.5 ng/mL TNF-α (PeproTech, Rocky Hill, NJ, USA) in 1% FBS/DMEM, or RAW-CM containing 1% FBS/DMEM for 24 h. Cultured supernatants were collected for inflammatory cytokine analysis by ELISA.

### 4.8. Statistical Analysis

Data were presented as mean ± standard error of the mean (SEM), and means were calculated using results from at least three independent experiments. Differences between groups were analyzed by one-way analysis of variance (ANOVA) and then followed by a Fisher’s least significant difference (LSD) test (IBM Statistical Product and Service Solutions (SPSS), version 19). The correlation between lunasin dosage and cytokine level was analyzed by Pearson’s correlation coefficient. Statistical significance was indicated by a *p*-value < 0.05.

## 5. Conclusions

Based on these results, this is the first finding that lunasin treatment might be beneficial in the inhibition of adipocyte inflammatory reactions through a decrease in IL-6 and MCP-1 secretions, and the retardation of obesity-related mediators that cause breast cancer cell metastasis, suggesting lunasin might block the cross-talk of these two cells. Furthermore, implementation of a lunasin-rich diet or dietary supplementation could be supported for the auxiliary prevention and/or therapy of obesity-related breast cancer.

## Figures and Tables

**Table 1:** Correlation of lunasin dosage and cytokine quantity.

Lunasin Versus Cytokine	IL-6	MCP-1
Correlation	−0.545	−0.861
*p*-Value	0.029	0.006

**Table 2:** Correlation of lunasin dosage and cytokine quantity.

Lunasin Versus Cytokine	IL-6	MCP-1
Correlation	0.026	−0.651
*p*-Value	0.926	0.006

## Supplementary Materials

Supplementary materials can be found at www.mdpi.com/1422-0067/17/12/2109/s1.

## Abbreviations

Ad-CM	adipocyte-conditioned medium
TNF-α	tumor necrosis factor
RAW-CM	RAW 264.7 cell-conditioned medium
VEGF	vascular endothelial growth factor
IL-6	interleukin-6
MCP-1	macrophage chemoattractant protein
LPS	lipopolysaccharide
NF-κB	nuclear factor-κB
ELISA	enzyme-linked immunosorbent assay
MTT	methylthiazole tetrazolium
EGF	epidermal growth factor
PPAR-γ	peroxisome proliferator-activated receptor-γ
LDL	low density lipoprotein
HMG	hepatic 3-hydroxy-3-methyl-glutaryl
