# Supplementary material for: Lunasin Attenuates Obesity-Associated Metastasis of 4T1 Breast Cancer Cell through Anti-Inflammatory Property
Source: Int J Mol Sci. 2016 Dec 15;17(12):2109. doi: 10.3390/ijms17122109 (PMC5187909; doi:10.3390/ijms17122109)
Supplement: Supplementary file 1 [file ijms-17-02109-s001.pdf]

Supplementary Materials: Lunasin Attenuates Obesity-Associated Metastasis of 4T1 Breast Cancer Cell through Anti-Inflammatory Property

Chia-Chien Hsieh, Chih-Hsuan Wang and Yu-Shan Huang

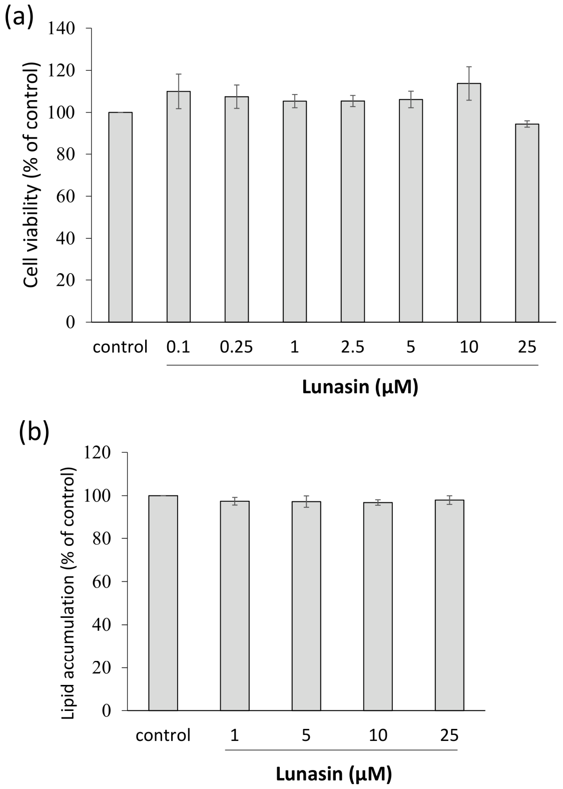

Figure S1. Lunasin did not affect 3T3-L1 fibroblast differentiation and fat storage in mature adipocytes. (**a**) Cell viability of cells treated with increasing doses of lunasin for 48 h measured by MTT test; (**b**) Lipid accumulation in 3T3-L1 cells was stained with Oil Red O, and lipid droplets were dissolved using 2-propanol, and then quantified by a spectrophotometer at 500 nm absorbance. Data are shown as mean ± SEM. Statistical analysis was tested by one-way ANOVA and then Fisher's LSD test.
